# Characterization of acrylic phantom for use in quality assurance of BNCT beam output procedure

**DOI:** 10.1093/jrr/rrae089

**Published:** 2024-11-19

**Authors:** Nishiki Matsubayashi, Naonori Hu, Takushi Takata, Akinori Sasaki, Hiroaki Kumada, Satoshi Nakamura, Akihiko Masuda, Hiroki Tanaka

**Affiliations:** Particle Radiation Oncology Research Center, Institute for Integrated Radiation and Nuclear Science, Kyoto University, 2, Asashiro-Nishi, Kumatori-cho, Sennan-gun, Osaka 590-0494, Japan; Particle Radiation Oncology Research Center, Institute for Integrated Radiation and Nuclear Science, Kyoto University, 2, Asashiro-Nishi, Kumatori-cho, Sennan-gun, Osaka 590-0494, Japan; Kansai BNCT Medical Center, Educational Foundation of Osaka Medical and Pharmaceutical University, Daigakumachi, Takatsuki, Osaka 569-0801, Japan; Particle Radiation Oncology Research Center, Institute for Integrated Radiation and Nuclear Science, Kyoto University, 2, Asashiro-Nishi, Kumatori-cho, Sennan-gun, Osaka 590-0494, Japan; Kansai BNCT Medical Center, Educational Foundation of Osaka Medical and Pharmaceutical University, Daigakumachi, Takatsuki, Osaka 569-0801, Japan; Institute of Medicine, University of Tsukuba, 1-1-1, Tennodai, Tsukuba, Ibaraki 305-8575, Japan; Division of Radiation Safety and Quality Assurance, National Cancer Center Hospital, 5-1-1 Tsukiji, Chuo-ku, Tokyo 104-0045, Japan; National Metrology Institute of Japan, National Institute of Advanced Industrial Science and Technology, 1-1-1 Umezono, Tsukuba, Ibaraki 305-8568, Japan; Particle Radiation Oncology Research Center, Institute for Integrated Radiation and Nuclear Science, Kyoto University, 2, Asashiro-Nishi, Kumatori-cho, Sennan-gun, Osaka 590-0494, Japan

**Keywords:** BNCT, neutron, gamma-ray, phantom, quality assurance

## Abstract

The accelerator-based boron neutron capture therapy (BNCT) system has been approved for specific cases covered by health insurance, and clinical trials for new cases in Japan are currently being conducted on other systems. Owing to the progress of accelerator-based BNCT, the operation of medical physics must be rendered more efficient. A water phantom is used for the quality assurance (QA) of the BNCT beam output procedure; however, a solid phantom is preferred for routine QA because of its ease of use. Additionally, because water phantoms cannot be readily used in some facilities owing to structural problems, solid phantoms are preferred for unified measurements at different facilities to compare beam outputs. In this study, we perform irradiation tests using an acrylic phantom and verify that an acrylic phantom can be used for QA. The distribution of thermal neutron flux and gamma-ray dose rate inside the acrylic phantom are evaluated through experiments and simulations. The results indicate that the acrylic phantom is suitable for routine QA and for comparing beam outputs among different systems. In the future, the same irradiation tests will be conducted at other facilities.

## INTRODUCTION

Boron neutron capture therapy (BNCT) is a type of radiotherapy based on nuclear reactions with a boron compound. It relies on the nuclear reaction of boron-10, which produces highly linear energy transfer particles (alpha particles and lithium nuclei) deposited as energy in human cells. Therefore, the reaction can provide the cells containing a boron compound with a therapeutic dose. Clinical BNCT has been conducted using reactor-based neutron sources; however, accelerator-based neutron sources have recently been used because of the progress in accelerator technology [1–3]. Accelerator-based BNCT can be conducted inside hospitals and has been recently considered as an alternative treatment to reactor-based BNCT [4, 5]. The first accelerator-based BNCT system used in clinical trials was developed by Sumitomo Heavy Industries in cooperation with Kyoto University in 2008 [6]. In this system, a 1-mA proton beam at 30 MeV accelerated by a cyclotron is irradiated onto a Be target to generate fast neutrons with energies up to 28 MeV. The fast neutrons emitted from the ^9^Be (p, n) ^9^B reaction pass through a moderator and shaper (known as a beam-shaping assembly, BSA) to form a spectrum suitable for BNCT, in which the neutron energy is reduced to epithermal neutrons. This system obtained approval for the manufacturing and sales of a new medical device from the Japanese Ministry of Health, Labor, and Welfare in March 2020, and was approved for reimbursement for unresectable, locally advanced, and recurrent carcinoma of the head and neck region covered by the Japanese national health insurance system in June 2020. At University of Tsukuba, an 8 MeV proton beam accelerated by a linear particle accelerator was irradiated to a Be target. For this system, the average proton current required to achieve an epithermal neutron flux for BNCT is 10 mA [7, 8]. A linear particle accelerator-based neutron source with an Li target was constructed at the National Cancer Center Hospital, where the neutron energy generated via a ^7^Li (p, n) ^7^Be reaction was 786 keV [9, 10]. For this system, 12 mA protons at 2.5 MeV were delivered to the target, and the target structure was designed conically for the cooling system. These systems are currently undergoing clinical trials for the treatment of new-onset glioblastomas, malignant melanomas and angiosarcomas.

The BNCT irradiation field contains high-intensity neutrons and gamma rays. To conduct an appropriate quality assurance (QA) of the BNCT beam output procedure, the distribution of neutrons and gamma -rays inside a phantom must be measured [11]. Measurements in a water phantom, which is a rectangular acrylic resin case filled with water, are the most widely performed type of measurement because the human body is composed primarily of water. As the number of facilities installing accelerator-based BNCT systems and insured clinical cases increases, the operation of medical physics, such as QA and quality control (QC), should be streamlined. However, setting up a water phantom correctly can be inconvenient because of the time required for filling, positioning and draining. An alternative to the water phantom is the solid phantom, which is preferred for routine QA to verify the correct beam properties prior to treatment owing to its ease of use and measurement speed [12]. Additionally, water phantom cannot be readily used at some facilities owing to structural problems. For example, because a BNCT system is constructed as a vertical beam, the air spaces in the surface layer inside the water phantom render it difficult to correctly measure the distributions. The use of a solid phantom is preferred for measurements at different facilities to compare the beam outputs among systems.

A treatment planning system based on Monte Carlo simulation is currently being used in BNCT to simulate radiation transport and calculate the dose delivered to a patient. Notably, the relationship between experimental and simulation results must be evaluated. To calculate neutrons from simulation, the thermal scattering law (TSL) of the phantom material should be considered. The TSL describes the manner by which the scattering of thermal neutrons changes in a moderator. In simulating thermal neutron transport, the appropriate scattering cross-section must be used because neutron scattering is sensitive to the atomic structure in the thermal-energy region [13, 14]. The TSL of acrylic has been obtained from ENDF/B-VIII.0 since 2018 and has been introduced in JENDL-5 [15, 16]. In this study, we investigated the suitability of acrylic phantoms for BNCT through experiments and simulations.

## MATERIALS AND METHODS

### Irradiation test using acrylic phantom

**Fig. 1:**
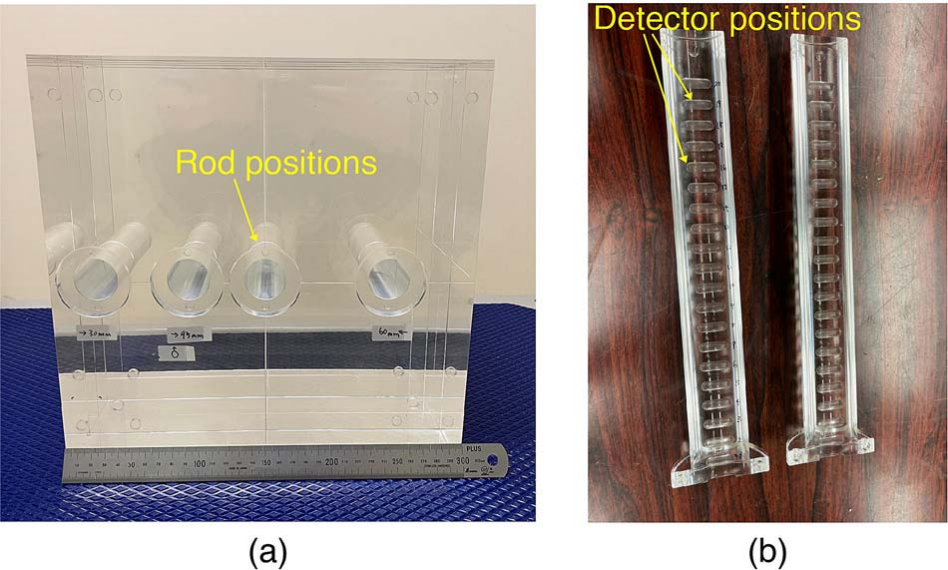
Photographs of irradiation test using PMMA phantom (**a**: PMMA phantom, **b**: PMMA rod for TLD).

The acrylic used in this study was polymethyl methacrylate (PMMA), and the acrylic phantom used in this study is referred to as the ‘PMMA phantom’. The PMMA phantom measured 30 × 30 × 30 cm and featured a density of 1.20 g/cm^3^, as shown in Fig. 1a. Figure 1b shows a cylindrical rod that can be inserted into the phantom, thus enabling detectors to be installed at different depths from the surface of the phantom. Several types of rods exist that correspond to the measurement methods. The rods corresponding to each measurement method contained spaces, which enables samples or dosimeters to be placed at specified positions inside the phantom.

In this study, the thermal neutron flux and gamma-ray dose inside the PMMA phantom were measured using the gold activation method and a gamma-ray dosimeter, respectively. The gold activation method was used to measure the thermal neutron flux, and thermo-luminescent dosimeter (TLD) and glass dosimeter were used as a gamma-ray dosimeter, because these measurement methods have been used for the QA of the BNCT [11, 17]. Irradiation tests were performed using a cyclotron-based epithermal neutron source at the Institute for Integrated Radiation and Nuclear Science, Kyoto University, which is the same system used with the approval for a medical device [6]. The irradiation tests were performed using a collimator with a diameter of 12 cm, which is typically used in BNCT [11, 18]. The phantom was installed at the center of the collimator, and the distributions of the thermal neutron flux and gamma-ray dose rate along the central beam were measured.

### Measurement method

To measure the thermal neutron flux, gold foils were placed along the central beam axis, with or without cadmium covers, in the PMMA phantom. For the gold foils without cadmium covers, PMMA capsules of the same size as the covers were used to reduce the detector-positioning error. The thermal neutron flux was measured using the activation method, in which the radioactivity of the activated gold sample after irradiation was measured using a Ge detector [18]. The thermal neutron fluxes were evaluated at depths of 0, 1, 2, 3, 4, 6, 9, 12 and 15 cm from the phantom surface.

The gamma-ray dose rate was measured using the TLD. The TLD was composed of BeO powder enclosed in a quartz glass capsule (UD-170LS, Panasonic), which exhibited low sensitivity to thermal neutrons. A thermal neutron correction factor was used to remove the thermal neutron sensitivity of the TLD [19]. To determine between the count measured by the TLD and gamma-ray dose value, calibration tests were performed in advance at the ^60^Co gamma-ray source [20]. The gamma-ray dose rate was evaluated at the same depth as in the thermal neutron flux measurement. In addition, the gamma-ray dose rate was evaluated using a glass dosimeter (GD-302 M, AGC Techno Glass). The glass dosimeter exhibited thermal neutron sensitivity, although its correction factor is not known, and was used with an LiF capsule to shield against thermal neutrons [17]. The LiF capsule was composed of LiF ceramic-enriched ^6^Li (95%); it featured a density of 2.4 g/cm^3^ and a thickness of 2 mm. The calibration tests of the glass dosimeter were performed as well as the TLD.

### Monte Carlo simulation

**Fig. 2:**
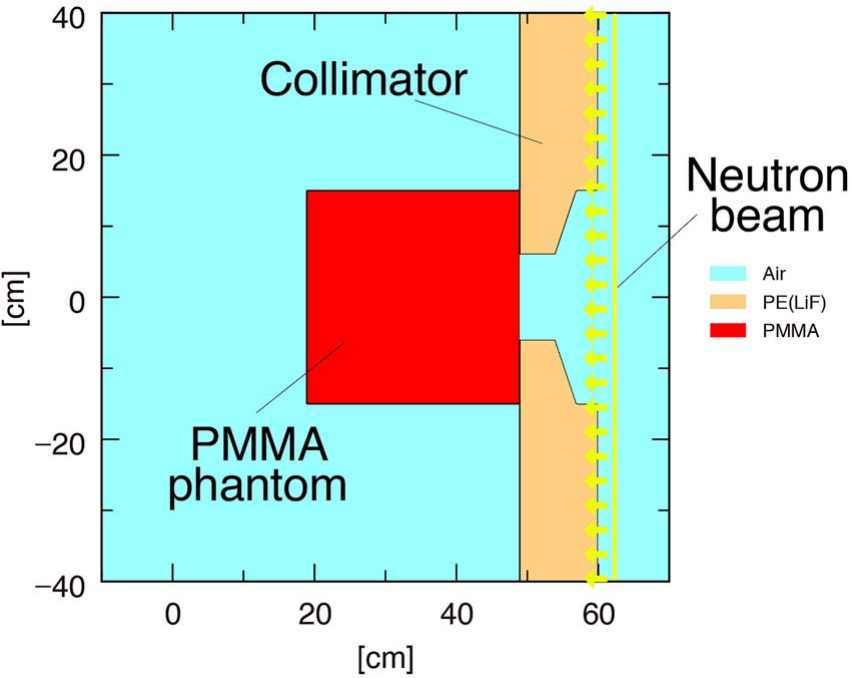
Schematic layout of irradiation test using PMMA phantom (PHITS ver. 3.28).

To safely implement BNCT, the simulated thermal neutron flux and gamma-ray dose rate inside the PMMA phantom were compared with experimental measurements as well as with the water phantom [18]. The Particle and Heavy Ion Transport Code System (PHITS, ver. 3.28) was used to simulate both neutron and photon transport in BNCT [21]. Irradiation tests on the PMMA phantom were simulated using the PHITS. Figure 2 shows a schematic illustration of the irradiation test with the PMMA phantom simulated by PHITS. The distributions of the thermal neutron flux and gamma-ray dose rate were measured inside the PMMA phantom, based on assuming that the gold foil and TLD does not perturb the beam. However, as the distributions of the glass dosimeters were affected by the LiF capsule, the shape of the capsules was simulated. Annular neutron and photon sources were set behind the collimator. The source strengths were calibrated to the experimental results by the thermal neutron flux and gamma-ray dose-rate distributions measured inside the water phantom so far. The TSL data of PMMA (Lucite) from JENDL-5 was used because JENDL is easy to use in PHITS [16]. Before the TSL of PMMA was obtained from ENDF/B-VIII.0, the facility that used the acrylic phantom substituted the TSL of acrylic with that of water or polyethylene. To evaluate these data, the effect of the TSL of the material on thermal-neutron-flux distribution was evaluated. Furthermore, to confirm the differences due to the nuclear data libraries, the thermal neutron flux distribution was evaluated using the TSL of ENDF/B-VIII.0, in addition to JENDL-5. The TSL from ENDF/B-VIII.0 was converted to an ACE file using FRENDY2.00 [22]. In this study, the thermal neutron distribution was simulated using the TSLs of PMMA (JENDL-5 and ENDF/B-VIII.0), water and polyethylene, to evaluate the effects of the different TSLs. When the TSL was changed, the density and volume of the phantom were maintained the same as those of the PMMA phantom to observe the effect of only the TSL.

## RESULTS

### Thermal neutron flux distribution

**Fig. 3:**
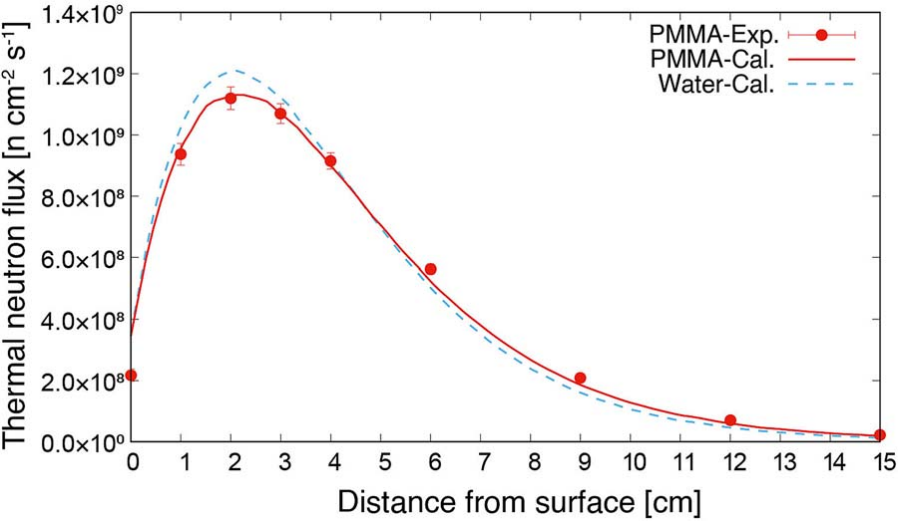
Thermal neutron flux distributions inside PMMA and water phantom.

The thermal neutron fluxes inside the PMMA phantom measured using the gold activation method (PMMA-Exp.) and simulated using the PHITS (PMMA-Cal.) are shown in Fig. 3. The measurement and simulation results indicated good agreement in the PMMA phantom. To compare the results with those of the water phantom, the only simulated thermal neutron distribution in the water phantom (Water-Cal.) is added to Fig. 3. Comparing the PMMA with the water phantom, the thermal neutron flux at a depth of 2 cm of the PMMA phantom was 7.6% lower than that of the water phantom, whereas it was 44% higher at 15 cm owing to the lower hydrogen density of PMMA compared with that of water. The results confirmed that the thermal neutron distribution differed depending on the phantom material; however, the difference was simulated correctly using the PHITS.

### Gamma-ray dose rate distribution

**Fig. 4:**
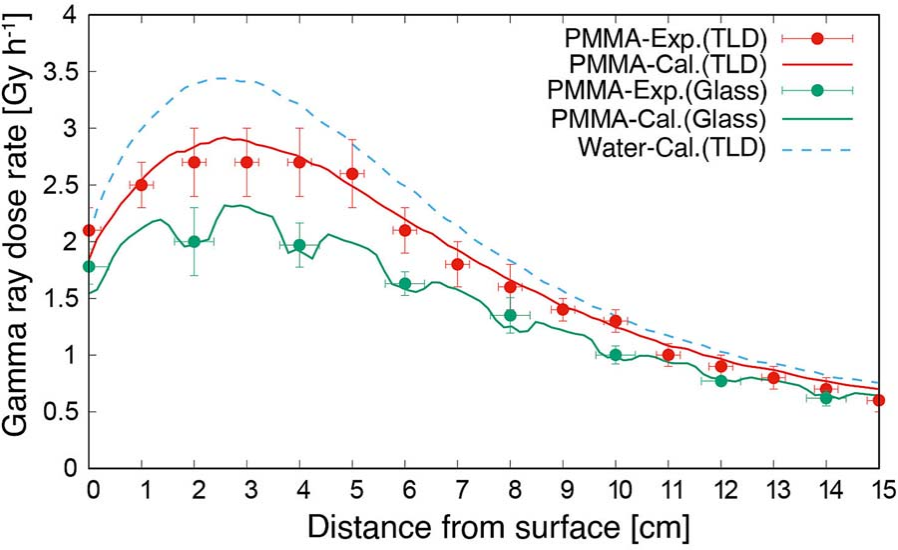
Gamma-ray dose rate distributions inside PMMA and water phantom.

The gamma-ray dose rate distributions inside the PMMA phantom, as measured using the TLD (PMMA-Exp.(TLD)) and simulated using the PHITS (PMMA-Cal.(TLD)), are shown in Fig. 4. The gamma-ray dose rate in the PMMA phantom was lower than that in the water phantom (Water-Cal.(TLD)). The experimental and simulation results showed good agreement. The gamma-ray dose rate indicated by the glass dosimeter with the LiF capsule (PMMA-Exp.(Glass)) decreased owing to the shielding thermal neutron flux. However, based on the LiF capsules simulated using the PHITS (PMMA-Cal.(Glass)), the simulated distribution was the same as that in the experiment. Comparing the PMMA-Exp.(TLD) and PMMA-Exp.(Glass), the dose rate with the LiF capsule was 18% lower than that without the capsule. Thus, if a glass dosimeter with an LiF capsule is used, then the size and density of the capsule would be simulated correctly.

### Thermal neutron scattering law

**Fig. 5:**
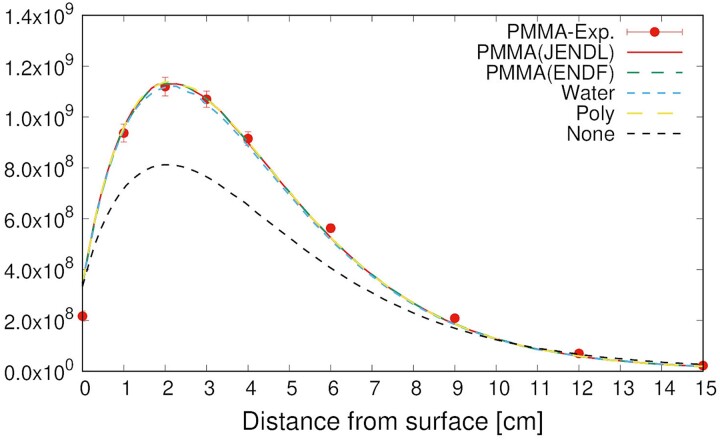
Thermal neutron flux distribution using TSLs of PMMA (JENDL and ENDF), water and polyethylene, and not considering TSL.

**Fig. 6:**
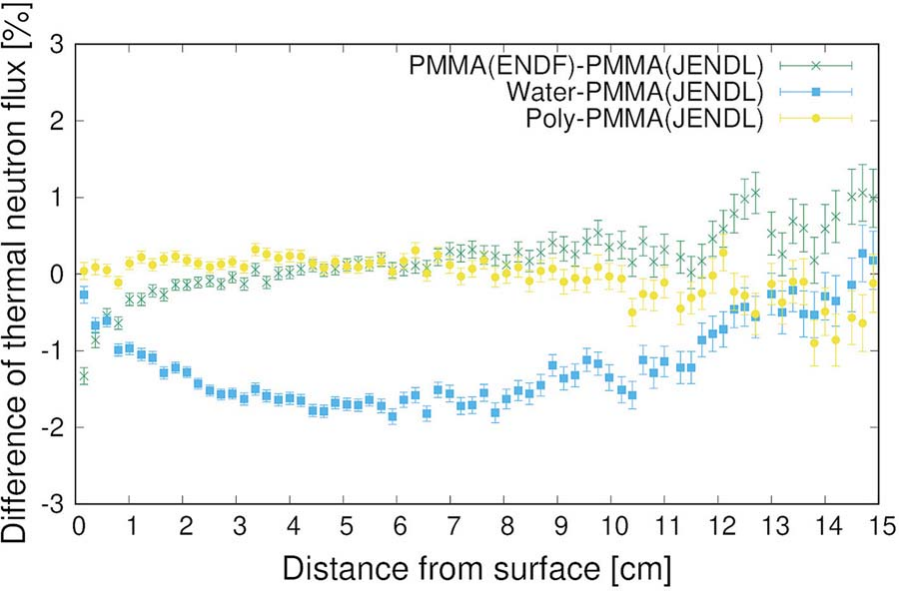
Subtraction of thermal neutron fluxes of PMMA (ENDF), water and polyethylene from that of PMMA (JENDL).

Figure 5 shows the differences in the thermal neutron distributions using the TSL of PMMA (JENDL and ENDF) with those of water and polyethylene (Poly), and not considering the TSL (None). First, the thermal neutron distribution without the TSL was much lower than that obtained experimentally; thus, the TSL must be considered in the simulation. Regardless of the TSL used, the values were within the measurement uncertainty range of the activation method. Upon observing the differences meticulously, no difference was indicated between PMMA and polyethylene, although the results are different from those of water due to the difference in hydrogen content. To observe the difference in the TSL in detail, Fig. 6 shows the subtraction of the thermal neutron fluxes of PMMA (ENDF), water and polyethylene, from that of PMMA (JENDL). Comparing the PMMA (JENDL) with water and polyethylene, the maximum difference for the case of water was 1.9% and that for polyethylene was less than 1%; these low differences are due to the fact that both PMMA and polyethylene are plastics. Additionally, the difference for the case of water increased at approximately 2 cm, where the neutron flux was higher and approached zero as the flux decreased. Comparing JENDL with ENDF, the thermal neutron flux of ENDF was 1.3% lower than that of JENDL near the phantom surface; the reason is not known. However, the difference was caused by conversion to the ACE file, because the TSLs of JENDL-5 were based on ENDF/B-VIII.0. The conversion software and its versions will be investigated in future studies. The thermal neutron flux distribution of ENDF was closer to that of the experiment, and we plan to use the TSL of ENDF when irradiation tests are conducted at other facilities.

## DISCUSSION

The thermal neutron fluxes and gamma-ray dose rates inside the PMMA phantom were evaluated through experiments and simulations. The distributions simulated by the PHITS agreed well with the experimental results, and the measurement method using the PMMA phantom is confirmed to be feasible for the QA of the BNCT beam output procedure. Considering its ease of use and versatility (independent of the structure of the irradiation system), the measurement method using the PMMA phantom is suitable for routine QA and comparing beam outputs among the systems. However, in measurements using a real-time detector, the detectors could not use a driving device to change their position inside the PMMA phantom without entering the room [23–25]. To measure the distribution inside the PMMA phantom using real-time detectors, several detectors that do not perturb the field must be installed at each depth. If different acrylic phantom is used at another facility for the QA of the BNCT procedure, it is necessary to consider the effects of the differences in density and composition of the phantom caused due to the manufacturers. When the density of the PMMA phantom was changed by ±0.05 g/cm^3^, the thermal neutron flux at 2 and 15 cm depth was changed by ∓3% and ± 15%, respectively. It was found that the difference of the density has a huge effect on the measurement of the deep position, and it is important to evaluate the accurate density of the PMMA phantom. Additionally, the effect of acrylic discoloration owing to long-term irradiation should be considered.

The difference in the thermal neutron distribution with TSL was evaluated using the PHITS. The maximum difference in the TSL between PMMA and water was approximately 2%. Because of the measurement uncertainties of the activation method, whether the TSL of PMMA or water corresponds to the experiments could not be determined. However, when the thermal neutron flux distribution in the PMMA phantom was simulated using the TSL of water, the neutron fluxes were simulated to be 1.3 and 1.9% lower than those obtained using the TSL of PMMA at 2 and 6 cm depths from the phantom surface, respectively. The stability of the thermal neutron flux can be measured without any issue; however, if the neutron source data, which were used in treatment planning, were created based on measured thermal neutron flux distribution inside the PMMA phantom and simulations using an inaccurate TSL, then the plan was adversely affected.

## CONCLUSION

Irradiation tests using a PMMA phantom were performed, and the distributions of the thermal neutron flux and gamma-ray dose rate were measured through experiments and simulations using the PHITS. The PMMA phantom might be promising for routine QA and performance comparison between facilities. In the future, the same irradiation tests will be conducted at different BNCT facilities.
